# Seasonal and temperature‐related movement of Colorado River cutthroat trout in a low‐elevation, Rocky Mountain stream

**DOI:** 10.1002/ece3.2847

**Published:** 2017-03-10

**Authors:** Brian W. Hodge, Kyle D. Battige, Kevin B. Rogers

**Affiliations:** ^1^ Trout UnlimitedSteamboat SpringsCOUSA; ^2^ Colorado Parks and WildlifeFort CollinsCOUSA; ^3^ Colorado Parks and WildlifeAquatic Research SectionSteamboat SpringsCOUSA

**Keywords:** cutthroat trout, distribution, habitat use, life history, movement, stream temperature

## Abstract

Mobile species will migrate considerable distances to find habitats suitable for meeting life history requirements, and stream‐dwelling salmonids are no exception. In April–October 2014, we used radio‐telemetry to examine habitat use and movement of 36 Colorado River cutthroat trout *Oncorhynchus clarkii pleuriticus* (CRCT) in a 14.9‐km fragment of Milk Creek, a relatively low‐elevation stream in the Rocky Mountains (Colorado). We also used a network of data loggers to track stream temperature across time and space. Our objectives were to (1) characterize distribution and movement of CRCT, (2) evaluate seasonal differences in distribution and movement of CRCT, and (3) explore the relationship between stream temperature and distribution and movement of CRCT. During the course of our study, median range of CRCT was 4.81 km (range = 0.14–10.94) and median total movement was 5.94 km (range = 0.14–26.02). Median location of CRCT was significantly further upstream in summer than in spring, whereas range and movement of CRCT were greater in spring than in summer. Twenty‐six of the 27 CRCT tracked through mid‐June displayed a potamodromous (freshwater migratory) life history, migrating 1.8–8.0 km upstream during the spring spawning season. Four of the seven CRCT tracked through July migrated >1.4 km in summer. CRCT selected relatively cool reaches during summer months, and early‐summer movement was positively correlated with mean stream temperature. Study fish occupied stream segments in spring and fall that were thermally unsuitable, if not lethal, to the species in summer. Although transmitter loss limited the scope of inference, our findings suggest that preferred habitat is a moving target in Milk Creek, and that CRCT move to occupy that target. Because mobile organisms move among complementary habitats and exploit seasonally‐unsuitable reaches, we recommend that spatial and temporal variability be accounted for in delineations of distributional boundaries.

## 1 Introduction

Mobile species will move to find habitats suitable for meeting life history requirements, and stream‐dwelling salmonids are no exception. Inland trout (e.g., *Oncorhynchus and Salmo* spp.) will migrate considerable distances to reach optimal spawning, foraging, and overwintering habitats (Brown & Mackay, 1995; Gowan & Fausch, 2002; Schoby & Keeley, 2011). Moreover, movement is imperative to fishes living in streams where complementary, or important, non‐substitutable habitats are at disparate locations (Schlosser, 1995). In theory, the greater the distance between complementary habitat patches, the greater the movement accrued by individuals over their lifetime (Dunning, Danielson, & Pulliam, 1992; Schlosser, 1995).

A number of studies show that stream‐dwelling salmonids will move in search of thermally suitable habitats (e.g., Hillyard & Keeley, 2012; Jakober, McMahon, Thurow, & Clancy, 1998; Kaeding, 1996). Kaeding (1996) observed that rainbow trout *O. mykiss* and brown trout *S. trutta* in the Firehole River (Wyoming) seek out cool tributaries and main‐stem refugia in summer months. Similarly, and consistent with “habitat complementation” theory (Dunning et al., 1992; Schlosser, 1995; White & Rahel, 2008), Petty, Hansburger, Huntsman, and Mazik (2012) observed that movement of brook trout *Salvelinus fontinalis* in an Appalachian river network (West Virginia) coincided with peak summer temperatures and was inversely related to the initial distance between individuals and coldwater habitat patches.

**Figure 1:**
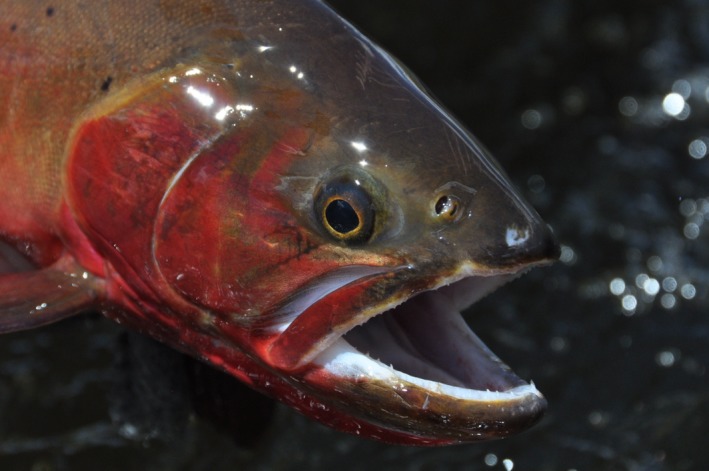
An adult Colorado River cutthroat trout *Oncorhynchus clarkii pleuriticus* (Photo by K. B. Rogers)

Like other inland cutthroat trout *O. clarkii,* Colorado River cutthroat trout *O. c. pleuriticus* (CRCT) persist in an increasingly fragmented landscape (Figure 1). While CRCT currently occupy 16% (5,200 of 32,300 km) of historical fluvial habitat in the upper Colorado River basin (Hirsch, Dare, & Albeke, 2013), 72% of populations are isolated above barriers in short (≤10 km), headwater segments. Historical conditions would have allowed for large‐scale movement among habitats and during different seasons and life history stages (Young, 2008). Today, only 5–6% of CRCT populations display migratory life histories (Hirsch et al., 2013).

Although a number of studies have reported on the habitat preferences and movement patterns of CRCT, few have evaluated factors influencing distribution and movement of CRCT. Research to date has addressed habitat use (Bozek & Rahel, 1991; Kershner, Bischoff, & Horan, 1997; Scarnecchia & Bergersen, 1986), temperature requirements (Roberts, Fausch, Peterson, & Hooten, 2013; Underwood, Myrick, & Rogers, 2012), and movement of CRCT (Young, 1996, 2011; Young, Rader, & Belish, 1997). However, to the best of our knowledge, only one natural‐setting study has examined seasonal differences in habitat use, distribution, and movement of CRCT (Young, 1998), and none have specifically examined effects of stream temperature on habitat use, distribution, and movement of CRCT (but see De Staso & Rahel, 1994). Young (1998) observed that range of CRCT did not differ between summer and autumn, whereas studies of other inland cutthroat trout sub‐species *O. clarkii* suggest that distribution and movement vary among seasons and also with stream temperature (Dobos, Corsi, Schill, DuPont, & Quist, 2016; Hilderbrand & Kershner, 2000; Hillyard & Keeley, 2012; Jakober et al., 1998). In this study, we (1) characterized the distribution and movement of CRCT, (2) evaluated seasonal differences in distribution and movement of CRCT, and (3) explored the relationship between stream temperature and distribution and movement of CRCT.

## 2 Methods

### 2.1 Study site

**Figure 2:**
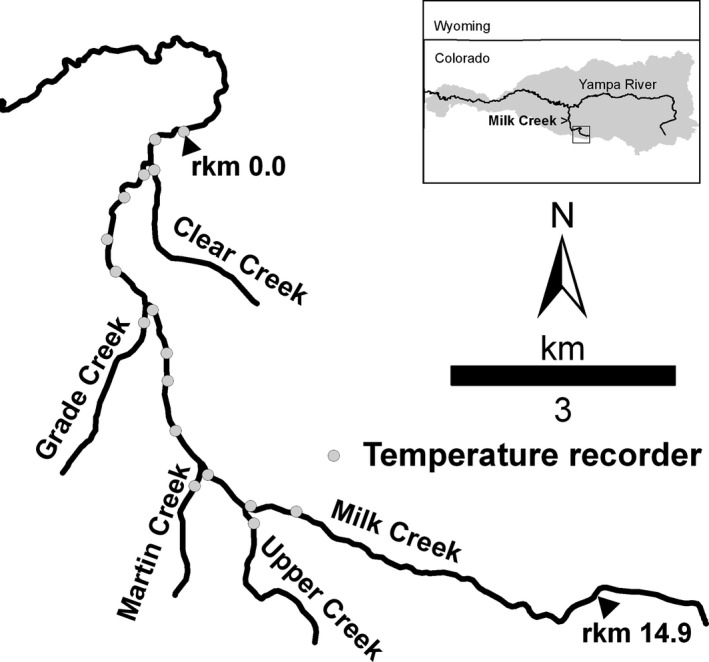
Locations of the study area at Milk Creek, Colorado, and of Milk Creek in the Yampa River basin (gray)

Milk Creek is a tributary to the Yampa River in northwest Colorado (watershed area = 578 km^2^; Figure 2). CRCT occupy the upstream‐most 14.9 km of Milk Creek (Hirsch, Albeke, & Nesler, 2006), hereafter the “study reach” (downstream limit = river km [rkm] 0.00; watershed area = 89 km^2^; elevation = 2,075–2,580 m above mean sea level). Bankfull discharge in the study reach is approximately 8.27 m^3^/s (Natural Resources Conservation Service, 2011), and summer discharge is typically <0.1 m^3^/s. Tributaries in the reach include Clear Creek (rkm 0.96), Grade Creek (rkm 4.29), Martin Creek (rkm 7.03), and Upper Creek (rkm 8.16). Other fishes in the reach include mountain sucker *Catostomus platyrhynchus*, mottled sculpin *Cottus bairdii*, speckled dace *Rhinichthys osculus*, and white sucker *Catostomus commersonii*.

Milk Creek and the CRCT therein serve as a compelling case study for several reasons. First, the CRCT population in Milk Creek occupies a relatively large, isolated fragment (≥100 rkm from other populations) and thus serves as a favorable subject for evaluating the spatial requirements and dynamics of a single population. Second, CRCT in Milk Creek possess a rare mitochondrial haplotype, which suggests they are of aboriginal origin (Rogers, Bestgen, & Epp, 2014) and likely candidates for retaining heritable components of life history (Martyniuk, Perry, Mogahadam, Ferguson, & Danzmann, 2003; Thériault, Garant, Bernatchez, & Dodson, 2007; Thrower, Hard, & Joyce, 2004). Last, CRCT in Milk Creek persist despite routine thermal challenges during summer. Extirpation of CRCT is likely to result in streams where the warmest weekly mean maximum temperature (MWMT) exceeds 26.0°C, and growth of CRCT declines or ceases when the maximum 30‐day average temperature (M30AT) exceeds 18.0°C (Roberts et al., 2013). In June–July of 2013, stream temperatures exceeded both of these criteria throughout a 4.0‐km segment of the CRCT population's purported range in Milk Creek, and in October of 2013, CRCT were captured throughout this segment.

### 2.2 Spatial referencing

Prior to conducting field work and collecting data, we used a combination of 1‐m‐resolution aerial imagery and sub‐meter resolution survey data to create a point shapefile depicting the study reach (downstream limit = rkm 0; ESRI ArcGIS 10.2). The shapefile, which consisted of more than 2,500 5‐m nodes, was loaded onto three GPS units for use throughout the term of the study. We found this approach to be simpler and less error prone than capturing coordinates in space and converting them to distances along the stream course.

### 2.3 Fish capture and tagging

Colorado River cutthroat trout were captured with backpack electrofishing gear on 15–16 April 2014 (rkm 0.0–5.5). CRCT were retained and placed in individual net pens labeled with the location of capture (±5 m). The first 36 CRCT > 55 g wet mass (range: mass = 56–178 g, total length = 185–266 mm) were anaesthetized for 5–8 min (mean = 6.3 min) with 30 mg/L Aqui‐S^®^ @ 20E (New Zealand Ltd., Lower Hutt, New Zealand; INAD #11‐741), adipose clipped, and implanted with a 1.2‐g radio transmitter cycling at 12 pulses per minute (battery life = 261 days; Advanced Telemetry Systems, Isanti, MN, USA). Transmitters were inserted through a small incision along the ventral wall and anterior to the pelvic girdle, and whip antennas exited between the pelvic girdle and vent. Incisions were closed with two or three #4‐0 catgut sutures. Fish were ventilated with stream water during the surgical procedure (mean duration = 4.5 min, range = 3.5–6.0 min), and recovered in individual buckets of stream water following surgery (mean duration = 7.6 min, range = 0.5–36.0 min). Once recovered, fish were released at original locations of capture.

### 2.4 Fish tracking

Foot‐based telemetry surveys were conducted approximately weekly from late‐April through mid‐October of 2014, and on 10 occasions between June 29 and July 15 (*n* = 30 total occasions). Crews typically divided into two one‐ or two‐person teams that covered upstream and downstream segments of the study reach. Surveys took from one to three full days to complete and covered 6.0–13.6 km of the study reach, depending on the number and dispersion of transmitter‐bearing CRCT at large. Surveys were extended into the four tributaries and downstream of the study reach when fish were detected in those locations. Transmitters were located using R4000 radio receivers coupled with three‐element Yagi antennas (Advanced Telemetry Systems). Once a transmitter was pinpointed to the nearest 5 m, transmitter number, location, date, and other notes were recorded. In addition, data were written on a small dry‐erase board and a GPS‐integrated camera was used to capture a photo of both the location site and location data. Because we did not create shapefiles for tributaries a priori, we used coordinates to mark fish locations in these streams and later snapped coordinates to survey‐based shapefiles. When transmitters were relocated in the same locations on two or more occasions, efforts were made to distinguish between live, transmitter‐bearing fish, mortalities, and expelled transmitters. The latter were removed from the streambed whenever possible. At the end of the season, locations, transmitter recoveries, and photographs were used to discriminate between sedentary, transmitter‐bearing fish and expelled transmitters, and between true movements and those derived from GPS error.

### 2.5 CRCT distribution and movement

We used three metrics to characterize CRCT distribution and movement in Milk Creek. Distance upstream (from rkm 0) served as a metric of individual location in the study area. Range, the distance between the upstream‐most and downstream‐most locations (Alexiades, Peacock, & Al‐Chokhachy, 2012; Gresswell & Hendricks, 2007; Young, 1996), served as a measure of an individuals’ travel corridor in a given time period. Finally, total movement, or the sum of all movements (Gresswell & Hendricks, 2007; Muhlfeld, Bennett, & Marotz, 2001; Young, 1996), served as a metric of individual activity in a given time period.

### 2.6 Seasonal differences in distribution and movement

To evaluate seasonal differences in distribution and movement, we compared CRCT locations, range, and total movement between seasons using one‐way Kruskal–Wallis tests. Because cutthroat trout have been shown to move in spring in association with spawning (Hilderbrand & Kershner, 2000; Schoby & Keeley, 2011; Young, 1996), in summer in association with increasing stream temperature (Dobos et al., 2016; Hillyard & Keeley, 2012), and in fall in association with declining stream temperature (Jakober et al., 1998), we used indicators of spawning activity (e.g., movement in and out of tributaries, behavior in tributaries) to distinguish between spring and summer and used temperature cues to distinguish between summer and fall (also see explanation under CRCT‐temperature relationships). Based on these methods, “spring” included the time period from April 15 to June 29, “summer” the time period from June 30 to August 25, and “fall” the time period from August 26 to October 13. All analyses were performed in R (R Core Team 2014) at α = 0.05.

### 2.7 Temperature monitoring

Stream temperature was monitored from mid‐May to mid‐October at 13 sites in the main‐stem and at one site in each of the four tributaries (from mid‐June to mid‐October at five of the sites; Figure 2). Data loggers (Onset Corporation, Bourne, MA, USA) recorded temperature (±0.2°C) every 15 min between the times of deployment and retrieval. Temperature metrics were calculated using WaTSS (Rogers, 2015).

### 2.8 CRCT–temperature relationships

We evaluated influences of stream temperature on CRCT distribution in Milk Creek through a two‐step process. First, we developed a temperature–occupancy relationship by fitting a logistic regression model in which the response variable was CRCT use (1 = occupied, 0 = available) and the predictor variable was mean daily temperature (Temp; °C). Because the thermal niches of salmonids are typically characterized by a curvilinear, or bell‐shared response (e.g., Al‐Chokhachy, Wenger, Isaak, & Kershner, 2013; Bear, McMahon, & Zale, 2007; Wenger et al., 2011), a quadratic term (Temp²) was also included as a predictor variable in the model. For this exercise, we combined the 17 temperature monitoring sites and adjacent segments into four reaches (mean length = 3.67 km). Each reach included three or more thermally similar (peak temperatures within 1°C) and spatially contiguous segments. The one exception was a reach comprised of Martin and Upper creeks, which were very similar to one another (and only to one another) with respect to temperature, but separated from one another by 1.0 km of main‐stem habitat. We used the svyglm function in the survey package (Lumley, 2004, 2014) in R (R Core Team 2014) to fit the model. Repeated measures from individual fish and differences in reach lengths (i.e., unequal nominal probabilities of use) were accounted for by nesting and weighting observations, respectively. The model was fit using data from only the period of June 30– August 25 both to ensure that the number of available reaches was equal to or greater than the number of transmitter‐bearing fish at large and to avoid capturing spawning‐related distribution shifts (three early‐season observations were omitted because an individual was still on or near its breeding grounds). Next, we used the temperature‐occupancy model to predict how the spatial bounds of the thermal niche might change with time. Specifically, we compared daily stream temperature data from all 17 segments of the study area to the empirically derived probability curve. A segment was classified as suitable on day *i* if the probability of use on day *i* was >0.5, and unsuitable on day *i* if the probability of use on day *i* was <0.5 (Al‐Chokhachy et al., 2013).

To test for evidence of temperature‐related movement among CRCT, we focused an analysis on the first 2 weeks of summer (June 30–July 14), during which stream temperatures spiked rapidly and telemetry surveys were conducted almost daily. We hypothesized that fish encountering unsuitably warm temperatures would move to cooler waters to thermoregulate and that fish encountering suitably cool temperatures would not. We tested this hypothesis by using the svyglm function in the survey package (Lumley, 2004, 2014) for R (R Core Team 2014) to fit another logistic regression model. The response variable was movement (>233 m; see Young, 1996) or lack thereof between event *i* and event *i* + 1 (1 = yes, 0 = no), and the predictor variable was mean daily temperature from the location at event i as determined from the nearest available temperature monitoring site. We recognized but could not account for the possibility that fish occupied local thermal refugia within a reach (Nielsen, Lisle, & Ozaki, 1994; Ebersole, Liss, & Frissell, 2001; Baird & Krueger, 2003; but see Schrank, Rahel, & Johnstone, 2003). Observations were nested within fish to account for repeated measures (i.e., fish was treated as a random effect). Three early‐season observations were omitted because an individual was still on or near its breeding grounds.

## 3 Results

### 3.1 Fish tracking

**Figure 3:**
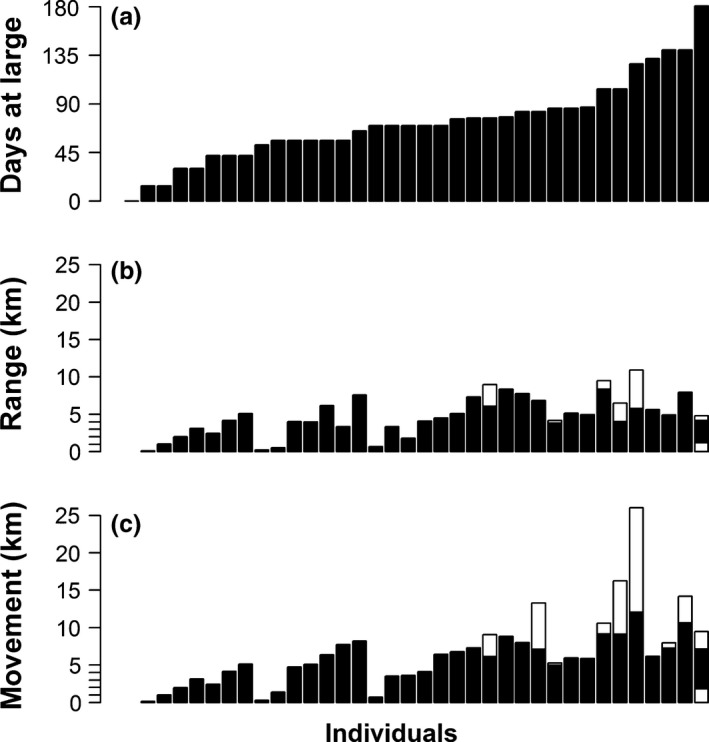
Days at large (a), range (b: black = spring, white = summer, gray = fall) and total movement (c: black = spring, white = summer, gray = fall) of transmitter‐bearing CRCT in Milk Creek. Each bar represents one individual (*n* = 36)

We tracked individuals for 0–181 days (mean = 73, *SE* = 6; Figure 3) and relocated individuals on 0–29 occasions (mean = 8, *SE* = 1). Detections of study fish decreased across the year in proportion to the number of transmitter‐bearing CRCT at large (*r* ^2^ = 0.94, *p* < 0.001). The number of transmitter‐bearing CRCT in the study decreased from 27 to 16 in the 2 to 3 weeks following the spawn (between mid‐ and late‐June). One fish was confirmed to be carrying a transmitter at the end of the study.

### 3.2 CRCT distribution and movement

During the course of our study, transmitter‐bearing CRCT used 90% of the purported range of the population. Observed locations of CRCT varied from 0.94 km downstream of the study reach to 12.48 km upstream in the study reach (median = 5.05 km upstream). CRCT were relocated as far as 1.05 km upstream in tributaries (median = 0.33 km upstream). Observed range of CRCT varied from 0.14 km to 10.94 km (median = 4.81 km), and total movement of CRCT varied from 0.14 km to 26.02 km (median = 5.94 km; Figure. 3). Range and movement increased with number of days at large (*p* ≤ 0.001, *r* ^2^ = 0.28–0.39) and number of relocations (*p* ≤ 0.008, *r* ^2^ = 0.17–0.34).

### 3.3 Seasonal differences in CRCT distribution and movement

Location, range, and total movement of CRCT differed between spring and summer (limited late‐season data prohibited rigorous comparisons between fall and the other two seasons). Spring locations varied from 0.94 km downstream to 9.83 km upstream, summer locations from 0.29 to 12.48 km upstream, and fall locations from 2.42 to 5.07 km upstream. Median location of CRCT was significantly further upstream in summer than in spring (8.38 km vs. 4.34 km; *n* = 52, *df* = 1, χ² = 14.492, *p* < 0.001), and only two of 16 fish tracked in both spring and summer had a more upstream median location in spring than in summer. Spring range varied from 0.14 km to 8.38 km, summer range from 0.00 to 10.78 km, and fall range was 1.22 km. Median range of CRCT was significantly larger in spring than in summer (4.17 km vs. 0.19 km; *n* = 48, *df* = 1, χ² = 12.037, *p* < 0.001), but two CRCT occupied larger ranges in summer than in spring (e.g., 10.78 km vs. 5.79 km). Spring movement varied from 0.14 to 12.05 km, summer movement from 0.00 to 13.97 km, and fall movement was 1.82 km. Median total movement of CRCT was significantly greater in spring than in summer (5.86 km vs. 0.42 km; *n* = 51, *df* = 1, χ² = 12.333, *p* < 0.001), but one individual displayed greater movement in summer than in spring (13.97 km vs. 12.05 km).

Most of the CRCT tracked for at least a month displayed one or more episodes of seasonal movement. Between mid‐April and mid‐June, 26 of 27 CRCT moved 1.81–7.95 km upstream in what were presumed to be spawning migrations. Median location increased from rkm 3.26 on April 29 to rkm 7.29 on June 10. Postspawn, seven of 26 CRCT migrated 1.81–5.79 km back downstream (one to within 5 m of its prespawn location), 15 remained in upstream locations, and four either died or expelled their transmitters. Of the 16 transmitter‐bearing CRCT at large at the beginning of summer (i.e., on June 30), nine remained within 200 m of their initial location, three migrated >200 m upstream (range = 0.30–7.98 km), one migrated >200 m (1.34 km) downstream, and three died or expelled their transmitters shortly thereafter. After mid‐July, movements >200 m were displayed only by three CRCT migrating downstream. In October, one transmitter‐bearing CRCT, and two that had expelled their transmitters, were recaptured below rkm 3.0 during a routine electrofishing survey.

Colorado River cutthroat trout used tributaries from late‐April to early‐July. One fish was observed in Clear Creek in late‐April and early‐May, and another fish was observed in Grade Creek in late‐May; both were subsequently observed in Upper Creek in June. On June 10, 17 of 27 CRCT occupied headwater tributaries: 14 were in Martin Creek and three were in Upper Creek. Three of the 14 CRCT observed in Martin Creek were subsequently observed in Upper Creek. Only one transmitter‐bearing CRCT was relocated in a tributary after June 24; that fish remained in Upper Creek until July 3. Overall, half of the CRCT tagged were observed in a tributary on one to six occasions.

### 3.4 Stream temperature

Temperatures in Milk Creek generally increased in May–June, peaked in July, and decreased in August–October. In June–September, daily means ranged from 7.0 to 21.3°C in the main stem and from 7.4 to 21.4°C in tributaries. Daily maxima ranged from 8.7° to 27.6° in the main stem and from 9.1° to 26.7° in tributaries. M30ATs ranged from 14.7 to 19.5°C, increased with distance downstream (*p* < 0.001, *r* ^2^ = 0.66), and were significantly higher in the main stem than in tributaries (18.0 ± 0.3 [mean ± *SE*] vs. 16.1 ± 0.8; ANOVA: *F*_1,15_ = 8.381, *p* = 0.011). M30ATs were 18.0–20.0°C in the downstream‐most 4.1 km of the study reach. MWMTs ranged from 19.1 to 26.2°C, increased with distance downstream (*p* < 0.001, *r* ^2^ = 0.84), and were significantly higher in the main stem than in tributaries (24.4 ± 0.40°C [mean ± *SE*] vs. 21.5 ± 1.2°C; ANOVA: *F*_1,15_ = 9.106, *p* = 0.009). MWMTs were ≥26.0°C in the downstream‐most 0.5–1.0 km of the study reach.

### 3.5 CRCT‐temperature relationships

**Figure 4:**
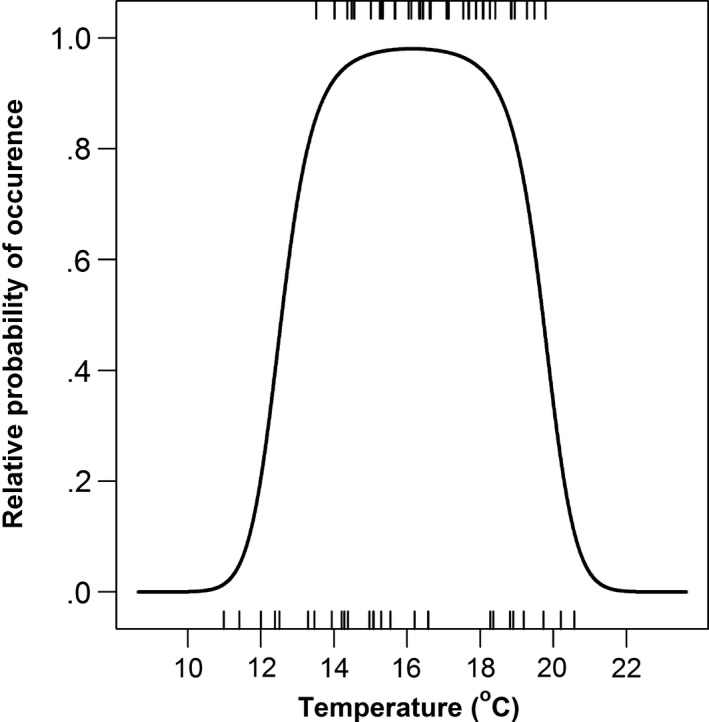
Observed (tick marks) and predicted (solid line) occupancy by CRCT versus mean daily stream temperature

**Figure 5:**
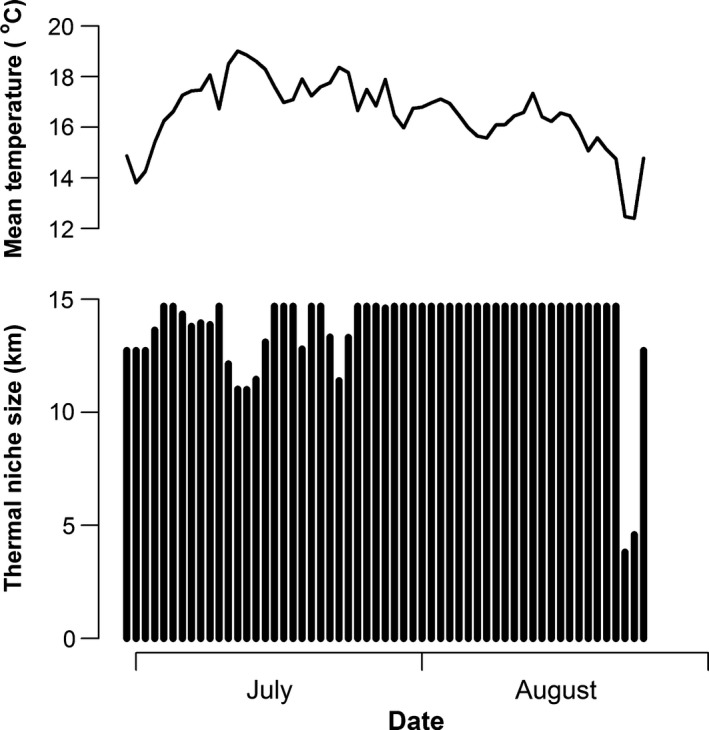
Overall mean stream temperature (top) and predicted extent of the summer (June 30–August 25) thermal niche for CRCT (bottom) versus date

Mean daily stream temperature was a significant predictor of CRCT occupancy in summer (*p* ≤ 0.002; Figure 4). The empirically derived temperature occupancy model suggested that the range of thermal suitability included areas where mean daily temperature was 12.6–19.7°C, and it predicted that the extent of suitable habitat varied (Figure 5). For example, the extent of the thermal niche was approximately 14.7 km throughout much of summer, but contracted approximately 3.7 km with peak temperatures in mid‐July (July 12–13; overall mean temperatures = 18.9–19.0°C).

**Figure 6:**
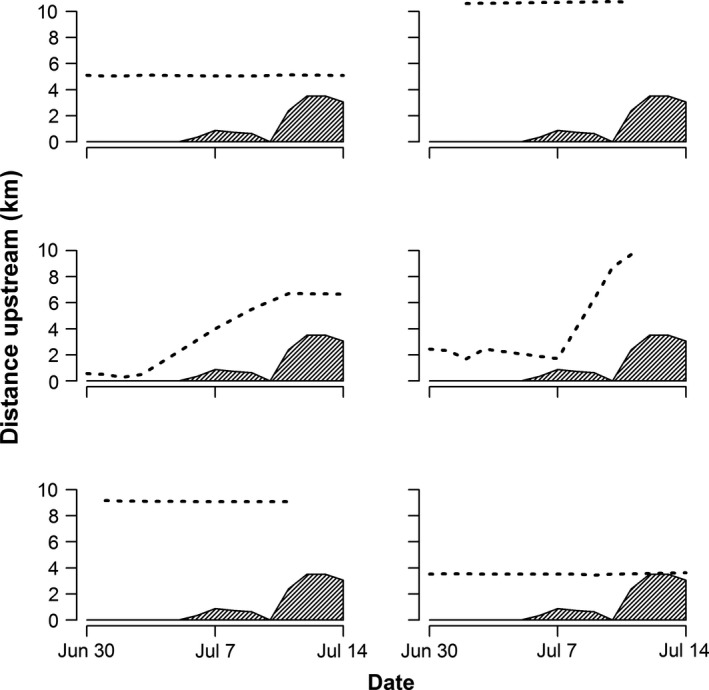
Location histories of six of the CRCT tracked from 30 June through 14 July 2014. Dashed lines depict individual locations through time and shaded areas depict the extent of steam that was too warm for CRCT (mean daily temperature >19.7°C)

Mean daily stream temperature was a significant predictor of fish movement (or lack thereof) during the first 2 weeks of summer (*p* = 0.035). Plots of fish locations versus time illustrated a pattern, whereby CRCT that entered summer in relatively warm locations below the downstream extent of the thermal niche moved during the first 2 weeks of summer (Figure 6). Conversely, fish that entered summer in relatively cool locations above the downstream extent of the thermal niche did not move.

## 4 Discussion

Our observations of CRCT movement exceeded those on record for CRCT (e.g., Hodge, Henderson, Rogers, & Battige, 2015; Young, 1996, 2011), but fell within the realm of expectations for inland cutthroat trout (e.g., Alexiades et al., 2012; Hilderbrand & Kershner, 2000; Schoby & Keeley, 2011). We observed that median range and total movement of CRCT in Milk Creek were 4.8 and 5.9 km, respectively. In other studies of CRCT, Young (1996) observed corresponding figures of 0.2 and 0.3 km in a Wyoming stream network (albeit over a shorter time period), and Young (2011) observed median maximum movements of 0.15–1.45 km in that same network (albeit over a longer time period). Hilderbrand and Kershner (2000) observed a median range of 1.4 km among the mobile faction of Bonneville cutthroat trout *O. c. utah* in a tributary to the Logan River (Utah), whereas Schoby and Keeley (2011) found that range of fluvial Westslope cutthroat trout *O. c. lewisi* in the Upper Salmon River (Idaho) varied from 6.8 to 235.9 km. It is interesting that we observed more movement by CRCT in Milk Creek than some have observed over longer time periods and of cutthroat trout in larger fluvial networks (e.g., Alexiades et al., 2012; Hilderbrand & Kershner, 2000; Young, 2011). Gowan, Young, Fausch, and Riley (1994) suggested fish movement may be relatively common in streams with variable and challenging environments.

Our findings regarding seasonal patterns of cutthroat trout movement align with and differ from other studies on the subject. We observed that CRCT moved more in spring and in association with spawning than in summer, but that CRCT moved as much as 14.0 km in summer. Young (1998), on the other hand, observed that movement of CRCT did not differ between summer and fall, and that CRCT moved <0.2 km in summer. Hilderbrand and Kershner (2000), Schmetterling (2001), and Schrank and Rahel (2004) all observed that cutthroat trout sub‐species moved greater distances in spring than in summer. However, none of these authors observed summer movements >0.7 km. Our results suggest that summer movements at Milk Creek might have been related to stream temperature.

Our empirically derived temperature‐occupancy curve for CRCT coincides with an established temperature‐growth relationship for the sub‐species (Brandt, 2009). We observed that probability of use by CRCT adults peaked around a mean daily temperature of 16.1°C, and Brandt (2009) found that growth of CRCT fry peaked at a rearing (i.e., mean) temperature of 15.3–16.4°C. This overlap might suggest that CRCT in Milk Creek find and select the reaches in which growth is optimal. However, because fish size can negatively influence the effect of temperature on salmonid growth and survival (i.e., large fish are less tolerant of warm temperatures; Selong, McMahon, Zale, & Barrows, 2001; Meeuwig, Dunham, Hayes, & Vinyard, 2004; Underwood et al., 2012; but see Recsetar, Ziegler, Ward, Bonar, & Caldwell, 2012), our observed peak of 16.1°C could be a conservative estimate of the populations’ optimum. Temperature‐acclimated CRCT fry (mean wet weight = 7.4 g, Brandt, 2009), for example, can tolerate a maximum temperature that is 1.3–2.2°C higher than CRCT adults (mean wet weight = 110.1 g; equation from Underwood et al., 2012). It follows that if use by adult CRCT in Milk Creek peaks at a mean temperature of 16.1°C, use by CRCT fry in Milk Creek could potentially peak at a mean temperature higher than 16.4°C. Meanwhile evidence from a laboratory experiment (Underwood et al., 2012) suggests thermal tolerance might vary among strains of CRCT. Namely, Underwood et al. (2012) found that CRCT from the relatively high‐elevation (el. 3,285 m) Lake Nanita population—the same brood stock used by Brandt (2009)—had a lower critical thermal maxima than CRCT from the relatively low‐elevation (el. 2,357 m) Trapper Creek population. Because the Milk Creek population is aboriginal and occupies a stream segment at 2,075–2,580 m above mean sea level, it is conceivable that CRCT from Milk Creek might, like Trapper Creek fish, be relatively tolerant of warm temperatures.

We observed three general patterns by which CRCT in Milk Creek tolerated and responded to elevated summer stream temperature, at least two of which have been observed before. Similar to Hillyard and Keeley (2012) and Petty et al. (2012), who observed Bonneville cutthroat trout and brook trout moving during the warmest part of summer, we observed a contingent of CRCT that moved from warm downstream reaches to relatively cool upstream reaches during a period of rapidly spiking stream temperatures. Also, like Burrell, Van Lear, and Dolloff (2000) and Schrank et al. (2003), who observed that brown trout and Bonneville cutthroat trout did not seek refuge in the face of threshold thermal conditions, we observed a contingent (represented by at least one individual in 2014) that elected not to move during the period of peak summer stream temperatures, despite occupying a warm downstream reach. Our findings are perhaps atypical in the respect that we observed both of these groups, as well as a third group that avoided a challenging summer thermal regime as a consequence of remaining upstream postspawn. Members of this third group appeared to have an advantage in 2014 because they expended the least amount of energy in exchange for the most benign summer thermal profile. Of course, any advantage or lack thereof would also depend on a number of other factors, including food availability and risk of predation. Because fish from all three groups spawned at the same time and in the same general locations, and thus presumably share a common gene pool, the variability in responses to stream temperature appears to be plastic.

One limitation of our study was the small sample size, owing to poor long‐term retention of transmitters. Transmitter loss could have occurred for a number of reasons. A likely explanation is that fish suffered from postspawning mortality, which can range from 13% to 89% in cutthroat trout (Gresswell, Liss, & Larson, 1994; Schmetterling, 2001; Vinyard & Winzeler, 2000). Of the 20 transmitters we recovered from either the streambed or a fish carcass, six were recovered within 1 week, and 12 within 2 weeks, of peak spawning activity. Our finding of a positive relationship between days at large and CRCT movement suggests the losses led to underestimation of range and movement. While premature transmitter and fish loss was limiting, it was not unique. Prior to completing their studies, Young (1996) lost 22 of 34 tag‐bearing CRCT to transmitter failure, predation, and other causes; Henderson, Kershner, and Toline (2000) lost 22 of 85 tag‐bearing trout to mortality and transmitter failure; and DeRito, Zale, and Shepard (2010) lost 54 of 164 tag‐bearing trout to predation and postsurgery (prespawn) mortality. Although transmitter loss limited our scope of inference, our study nevertheless advances knowledge about distribution and movement of CRCT.

In accordance with life history theory (e.g., Gross, 1987, 1996; Hendry, Brolin, Jonsson, & Berg, 2004), CRCT in Milk Creek should move among habitats when doing so will confer a fitness benefit. By moving to and spending October–April in relatively warm, downstream locations, CRCT in Milk Creek might enhance fitness by increasing both overwinter survival (Smith & Griffith, 1994) and spring growth (which is optimized in cutthroat trout at 9.5–18.0°C; Bear et al., 2007; Brandt, 2009; Ziegler et al., 2013). Increased growth and size at breeding allow for greater fecundity and egg size in female salmonids (Downs, White, & Shepard, 1997; Hodge, Wilzbach, & Duffy, 2014; Jonsson & Jonsson, 1999). By moving into and spawning in Martin and Upper creeks, where median particle size is closer to the ideal size of 10–30 mm (Schmetterling, 2000; Thurow & King, 1994; Young, 2008), and where there is less fine sediment than in Milk Creek (B. W. Hodge and K. D. Battige, personal observation), CRCT might increase survival of fertilized eggs (Holtby & Healey, 1986; Jensen, Steel, Fullerton, & Pess, 2009; Reiser & White, 1988). Finally, by moving upstream during periods of peak summer stream temperatures, CRCT might increase growth and over‐summer survival (Roberts et al., 2013). We conclude that preferred habitat is a moving target in Milk Creek and suspect that CRCT are required to move among habitats to optimize fitness.

This study contributes to a framework for understanding habitat use by mobile species. To the best of our knowledge, it is the first to examine how stream temperature influences distribution and movement of CRCT in a natural setting, and is among the first to examine seasonal differences in CRCT distribution and movement. Our findings have substantial management implications, both specifically with respect to inland trout and more generally with respect to other mobile organisms.

First, our results reinforce the notion that long‐range trout movements are more common than previously acknowledged (Gowan & Fausch, 1996; Gowan et al., 1994; Young, 2011). For example, as of 2010, migratory life histories had been documented in only 20 of 361 CRCT conservation (>90% genetically pure; UDWR (Utah Division of Wildlife Resources), 2000) populations (Hirsch et al., 2013). While we expected to observe movement of CRCT in Milk Creek, we were surprised that 96% of study fish displayed a potamodromous (freshwater migratory; Northcote, 1997) life history. We suspect that additional telemetry studies would reveal additional expressions of migratory life histories.

Second, our results offer a cautionary tale about the risks of using only seasonal data to evaluate habitat suitability and occupancy. Summer stream temperature and fish survey data from 2014 would suggest that CRCT are absent both from the downstream‐most 3.7 km of the purported range and from the upper tributaries of Milk Creek. Nevertheless, 14 of the 36 study fish were captured in the lower main stem in April, and 17 of 26 were relocated in Martin and Upper creeks in June. Meanwhile, the prospect of climate change has elevated the importance of stream temperature in evaluations of cutthroat trout status (Al‐Chokhachy, Alder, Hostetler, Gresswell, & Shepard, 2013; Wenger et al., 2011; Williams, Haak, Neville, & Colyer, 2009). For example, in the 2014 US Fish and Wildlife Service listing decision for Rio Grande cutthroat trout *O. c. virginalis*, an MWMT of 25°C was identified as the temperature threshold above which Rio Grande cutthroat trout populations could not persist (USFWS, 2014). Also, in a prior finding, populations were classified as too small to persist and were dismissed from further consideration, if they occupied segments shorter than 9.6 km (USFWS, 2008). Application of a 25°C threshold at Milk Creek would render a significant portion (as much as 5.8 km in 2013) of cutthroat trout‐bearing habitat “unsuitable,” and potentially lead one to remove the downstream‐most 5.8 km from the reported length of occupied habitat. This reduction would bring the occupied total stream length down to 9.1 km, and by the length criterion outlined in the 2008 listing decision, eliminate the Milk Creek CRCT population from consideration as a viable conservation unit. In the case of Milk Creek, one or more years of electrofishing and telemetry data were required to determine that seasonally‐unsuitable reaches were seasonally occupied by CRCT. Spatially and temporally restricted snapshots of stream temperature and fish distribution would have been misleading, and potentially led to undervaluation of the Milk Creek population.

Because Milk Creek is not the only stream where salmonids move among complementary habitats, and salmonids are not the only group of species to move among complementary habitats (Charbonnier et al., 2016; Dulaurent et al., 2011; Pope, Fahrig, & Merriam, 2000), we recommend that spatial and temporal variability of populations be accounted for in delineations of distributional boundaries. The alternative could result in inadvertent fragmentation of habitats (e.g., from poor placement of barriers) and failure to recognize and protect viable populations, including those that display migratory tendencies and/or harbor remnants of unique genetic diversity. Moreover, improving habitat connectivity and conserving genetic and life history diversity are important steps toward species conservation, especially in the face of a changing climate (Homel, Gresswell, & Kershner, 2015; Moore, Yeakel, Peard, Lough, & Beere, 2014; Rieman & Isaak, 2010; Roberts et al., 2013).

## Conflict of Interest

None declared.
